# Identification of new autoantibody specificities directed at proteins involved in the transforming growth factor β pathway in patients with systemic sclerosis

**DOI:** 10.1186/ar3336

**Published:** 2011-05-13

**Authors:** Guillaume Bussone, Hanadi Dib, Mathieu C Tamby, Cedric Broussard, Christian Federici, Geneviève Woimant, Luc Camoin, Loïc Guillevin, Luc Mouthon

**Affiliations:** 1 Institut Cochin, Université Paris Descartes, CNRS UMR 8104, 8 rue Méchain, F-75014 Paris, France; 2 INSERM U1016, 8 rue Méchain, F-75014 Paris, France; 3 Institut Cochin, Plate-forme Protéomique de l'Université Paris Descartes, CNRS UMR 8104, 22 rue Méchain, F-75014 Paris, France; 4 Etablissement Français du Sang, hôpital Saint-Vincent de Paul, Assistance Publique-Hôpitaux de Paris, 82 avenue Denfert-Rochereau, F-75674 Paris Cedex 14, France; 5 Université Paris Descartes, Faculté de Médecine, pôle de Médecine Interne et Centre de référence pour les vascularites nécrosantes et la sclérodermie systémique, hôpital Cochin, Assistance Publique-Hôpitaux de Paris, 27 rue du Faubourg Saint-Jacques, F-75679 Paris Cedex 14, France

## Abstract

**Introduction:**

Antinuclear antibodies (ANAs), usually detected by indirect immunofluorescence on HEp-2 cells, are identified in 90% of patients with systemic sclerosis (SSc). Thus, approximately 10% of SSc patients have no routinely detectable autoantibodies, and for 20% to 40% of those with detectable ANAs, the ANAs do not have identified specificity (unidentified ANAs). In this work, we aimed to identify new target autoantigens in SSc patients.

**Methods:**

Using a proteomic approach combining two-dimensional electrophoresis and immunoblotting with HEp-2 cell total and enriched nuclear protein extracts as sources of autoantigens, we systematically analysed autoantibodies in SSc patients. Sera from 45 SSc patients were tested in 15 pools from groups of three patients with the same phenotype. A sera pool from 12 healthy individuals was used as a control. Proteins of interest were identified by mass spectrometry and analysed using Pathway Studio software.

**Results:**

We identified 974 and 832 protein spots in HEp-2 cell total and enriched nuclear protein extracts, respectively. Interestingly, α-enolase was recognised by immunoglobulin G (IgG) from all pools of patients in both extracts. Fourteen and four proteins were recognised by IgG from at least 75% of the 15 pools in total and enriched nuclear protein extracts, respectively, whereas 15 protein spots were specifically recognised by IgG from at least four of the ten pools from patients with unidentified ANAs. The IgG intensity for a number of antigens was higher in sera from patients than in sera from healthy controls. These antigens included triosephosphate isomerase, superoxide dismutase mitochondrial precursor, heterogeneous nuclear ribonucleoprotein L and lamin A/C. In addition, peroxiredoxin 2, cofilin 1 and calreticulin were specifically recognised by sera from phenotypic subsets of patients with unidentified ANAs. Interestingly, several identified target antigens were involved in the transforming growth factor β pathway.

**Conclusions:**

We identified several new target antigens shared among patients with SSc or specific to a given phenotype. The specification of new autoantibodies could help in understanding the pathophysiology of SSc. Moreover, these autoantibodies could represent new diagnostic and/or prognostic markers for SSc.

## Introduction

Systemic sclerosis (SSc) is a connective tissue disorder characterised by excessive collagen deposition in the dermis and internal organs, vascular hyperreactivity and obliteration phenomena [1]. A large number of autoantibodies have been identified in the sera of SSc patients. Antinuclear antibodies (ANAs), usually detected by indirect immunofluorescence on HEp-2 cells, are identified in 90% of patients [2]. Some of them are disease-specific and mutually exclusive: anticentromere antibodies (ACAs), associated with limited cutaneous SSc (lcSSc) and possibly pulmonary arterial hypertension (PAH); anti-topoisomerase I antibodies (ATAs), associated with diffuse cutaneous SSc (dcSSc) and interstitial lung disease (ILD); and anti-RNA polymerase III antibodies, associated with dcSSc and scleroderma renal crisis (SRC) [3]. In addition, other autoantibodies have been found in the sera of SSc patients and include antifibrillarin, antifibrillin 1, anti-Th/To, anti-PM/Scl [3], antifibroblast [4-6] and anti-endothelial cell antibodies [7-9]. Overall, the only specific autoantibodies routinely tested for in SSc patients are ACAs, ATAs and, more recently, anti-RNA polymerase III antibodies.

Thus, approximately 10% of SSc patients have no routinely detectable autoantibodies, and for 20% to 40% of those with detectable ANAs, the nuclear target antigens of these ANAs have not been identified [2]. Therefore, further work is warranted to better determine the disease subset and prognosis for these patients. The specification of new autoantibodies could help in understanding the pathophysiology of SSc and reveal new diagnostic and/or prognostic markers.

Using a proteomic approach combining two-dimensional electrophoresis (2-DE) and immunoblotting, we recently identified target antigens of antifibroblast antibodies in patients with PAH [10]. In this work, using a similar proteomic approach with total and enriched nuclear protein extracts of HEp-2 cells as sources of autoantigens, we systematically analysed autoantibodies in SSc patients and identified a number of new target antigens for these autoantibodies.

## Materials and methods

### Immunoglobulin sources

Sera were obtained from 45 patients who fulfilled the LeRoy and Medsger criteria and/or the American Rheumatism Association criteria for the diagnosis of SSc. Sera were tested in 15 pools from groups of three patients with the same phenotype as described previously [10]. Four pools were from patients with identified ANAs (that is, ACAs, ATAs or anti-RNA polymerase III antibodies), ten pools were from patients with unidentified ANAs, and one pool was from patients without ANAs (Table 1). The sera from three patients with anti-RNA polymerase III antibodies who had experienced SRC were included in one of the two pools from patients with SRC. ANAs and ACAs were investigated by indirect immunofluorescence on HEp-2 cells; ACAs were characterised by a centromere pattern; ATAs and anti-RNA polymerase III antibodies were detected by using an enzyme-linked immunosorbent assay (ELISA) kit (INOVA Diagnostics, San Diego, CA, USA).

**Table 1:** Characteristics of pools of sera used as sources of IgG^a^

Main clinical characteristics	Autoimmunity	**Number of pools tested**^**b**^
Healthy blood donors	No ANA	1
dcSSc		
No visceral involvement	No ANA	1
Interstitial lung disease	ATA	1
Scleroderma renal crisis	Anti-RNA-pol III Abs	1
lcSSc		
Pulmonary arterial hypertension	ACA	1
No visceral involvement	ACA	1
dcSSc		
Scleroderma renal crisis	ANA with unidentified specificity	1
Pulmonary arterial hypertension	ANA with unidentified specificity	1
Interstitial lung disease	ANA with unidentified specificity	2
No visceral involvement	ANA with unidentified specificity	1
lcSSc		
Digital ulcers	ANA with unidentified specificity	1
Pulmonary arterial hypertension	ANA with unidentified specificity	1
Interstitial lung disease	ANA with unidentified specificity	1
No visceral involvement	ANA with unidentified specificity	2

^a^Abs: antibodies; ACA: anticentromere antibody; ANA: antinuclear antibody; anti-RNA-pol III Abs: anti-RNA polymerase III antibodies; ATA: antitopoisomerase I antibody; dcSSc: diffuse cutaneous systemic sclerosis; lcSSc: limited cutaneous systemic sclerosis; SSc: systemic sclerosis. ^b^A pool of sera from 12 healthy blood donors was tested as a control. Immunoglobulin G reactivities were tested in pools of three sera from patients with the same phenotype of SSc.

We used a pool of sera from 12 healthy blood donors as a control. Healthy controls (HCs) had no detectable disease, no remarkable medical history and no ANAs and were not taking any medication at the time of blood sampling. Serum samples were stored in aliquots at -80°C.

All patients and HCs gave their written informed consent according to the policies of the ethics committee of Cochin Hospital. They were included in the Hypertension Artérielle Pulmonaire (HTAP)-Ig study (Investigation and Clinical Research's contract 2005, CIRC 05066, promoter Assistance Publique-Hôpitaux de Paris).

### HEp-2 cell culture

HEp-2 cells, a cell line derived from a human laryngeal carcinoma, were obtained from EuroBio (Les Ulis, France) and cultured as described previously [8]. When confluent, the cells were detached by use of 0.05% trypsin-ethylenediaminetetraacetic acid (EDTA) (Invitrogen, Carlsbad, CA, USA).

### Protein extraction

Total proteins were extracted from HEp-2 cells as described previously [11]. Briefly, HEp-2 cells were suspended in a sample solution extraction kit (Bio-Rad Laboratories, Hercules, CA, USA) containing 2% (wt/vol) sulfobetaine zwitterionic detergent (SB 3-10) and the carrier ampholyte Bio-Lyte 3/10 (Bio-Rad Laboratories). Cell samples were sonicated on ice, and the supernatant was collected after ultracentrifugation. Finally, after protein quantification [12], 64 mM dithiothreitol (Sigma-Aldrich, St. Louis, MO, USA) was added, and the supernatant was aliquoted and stored at -80°C.

A protein extract enriched in nuclear proteins was obtained as previously described [13], which is referred to hereinafter as enriched nuclear protein extract. Briefly, HEp-2 cells were suspended in a buffer containing 10 mM 4-(2-hydroxyethyl)-1-piperazineethanesulfonic acid (HEPES), pH 7.9, 10 mM KCl, 0.1 mM EDTA, 0.1 mM ethyleneglycoltetraacetic acid (EGTA), 1 mM dithiothreitol and antiproteases. After incubation for 15 minutes on ice, 10% Nonidet P-40 (Sigma-Aldrich) was added and cells were vortexed. Cells were then resuspended, incubated for 15 minutes on ice and regularly vortexed in a buffer containing 20 mM HEPES, pH 7.9, 0.4 M NaCl, 1 mM EDTA, 1 mM EGTA, 1 mM dithiothreitol and antiproteases. After ultracentrifugation, the supernatant was washed in a precooled (-20°C) solution of 10% trichloroacetic acid in acetone with 0.07% 2-mercaptoethanol (Sigma-Aldrich) to eliminate salts as described previously [13]. Proteins were resuspended in the sample solution extraction kit and then quantified [12]. Finally, 64 mM dithiothreitol was added, and the sample was aliquoted and stored at -80°C.

### Two-dimensional electrophoresis

**Figure 1:**
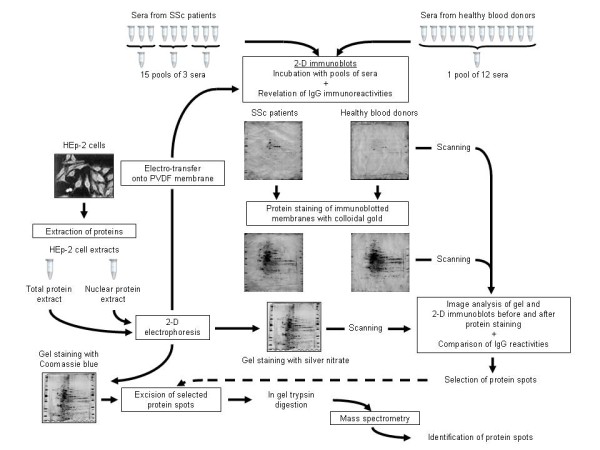
**Experimental design for screening anti-HEp-2 cell antibodies and identifying target autoantigens in SSc patients**. HEp-2 cell proteins were extracted and separated on two-dimensional (2-D) gels. Total and enriched nuclear protein extracts were used as substrates for 2-D electrophoresis. One gel was stained with silver nitrate and used as the reference gel, and proteins of the 11 other gels were transferred onto polyvinylidene difluoride (PVDF) membranes. Membranes were immunoblotted at 1:100 dilution with pooled sera from 12 healthy blood donors or from sets of three patients with the same phenotype of systemic sclerosis (SSc). After immunoglobulin G (IgG) immunoreactivities were revealed, the 2-D immunoblots were stained with colloidal gold to visualize the transferred proteins. 2-D immunoblots were scanned before and after colloidal gold staining with the use of a densitometer, then analysed by using image analysis software, and finally compared with the reference gel. Selected protein spots were extracted from another gel stained with Coomassie brilliant blue, and candidate proteins were identified by mass spectrometry. Database searching was used to identify the antigens.

The study protocol is depicted in Figure 1. We used a pH range of 3.0 to 10.0 and an acrylamide gradient of 7% to 18%, which allowed us to study a wide range of antigens of 10 to 250 kDa [11,14]. Proteins were isoelectrofocused with 17-cm immobilised pH gradient strips on the Protean IEF Cell System (Bio-Rad Laboratories) as described previously [11]. Thus, 100 μg of HEp-2 cell proteins from total or enriched nuclear protein extracts were loaded onto each strip. Before the second dimension, the strips were equilibrated and then proteins were transferred to gels as described previously [11,13]. Finally, one gel was stained with ammoniacal silver nitrate to serve as a reference for analysis of 2-D immunoblots [14].

### Electrotransfer and immunoblotting

After migration, proteins were transferred onto polyvinylidene difluoride membranes (Millipore, Billerica, MA, USA) by semidry transfer (Bio-Rad Laboratories) at 320 mA for 90 minutes. After being blocked, membranes were incubated overnight at 4°C with each of the sera pools from HCs and patients at a 1:100 dilution. Immunoglobulin G (IgG) immunoreactivities were revealed as described previously [11]. Specific reactivities were determined by densitometrically scanning the membranes (GS-800 calibrated densitometer; Bio-Rad Laboratories) with Quantity One software (Bio-Rad Laboratories). The membranes were then stained with colloidal gold (Protogold; British Biocell International, Cardiff, UK) and underwent secondary densitometric analysis to record labelled protein spots for each membrane.

Images of the reference gel and membranes were acquired by using the GS-800 calibrated densitometer and were analysed by using ImageMaster 2D Platinum 6.0 software (GE Healthcare, Buckinghamshire, UK) as described previously [11].

### In-gel trypsin digestion

Relevant spots were selected by comparing the 2-D immunoblots with the silver-stained reference gel and then extracted from another gel stained with Coomassie brilliant blue (Sigma-Aldrich). In-gel digestion involved the use of trypsin as described previously [13], and for all steps a Freedom EVO 100 digester/spotter robot was used (Tecan, Männedorf, Switzerland).

### Protein identification by mass spectrometry

Protein identification involved the use of a matrix-assisted laser desorption/ionization time of flight (MALDI-TOF)-TOF 4800 mass spectrometer (Applied Biosystems, Foster City, CA, USA) as previously reported [13]. Database searching involved the use of Mascot 2.2 software (Matrix Science, London, UK) and the GPS Explorer version 3.6 program (Applied Biosystems) to combine mass spectrometry (MS) and tandem mass spectrometry (MS/MS) queries of human proteins from the Swiss-Prot database [15].

### Biological network analysis

Protein lists of interest were analysed using Pathway Studio software (Ariadne, Rockville, MD, USA) [16]. Pathway Studio is a pathway analysis tool that uses automated text-mining engines to extract information from the literature. Briefly, protein lists were run against ResNet 7.0, a database of biological relations, ontologies and pathways. ResNet 7.0 covers human, mouse and rat proteins. The filters applied included "all shortest paths between selected entities" and "expand pathway". The information received was narrowed down to our protein lists to obtain their relationships. Protein entities belonging to different functional groups were represented as different shapes.

### Statistical analysis

Data are presented as mean values ± standard deviation. Positive identification of proteins by MALDI-TOF-TOF was based on a statistically significant Mascot score (*P *< 0.05). For peptides matching multiple members of a protein family, the reported protein is the one with the highest number of peptide matches.

## Results

### Analysis of HEp-2 cell proteomes

**Figure 2:**
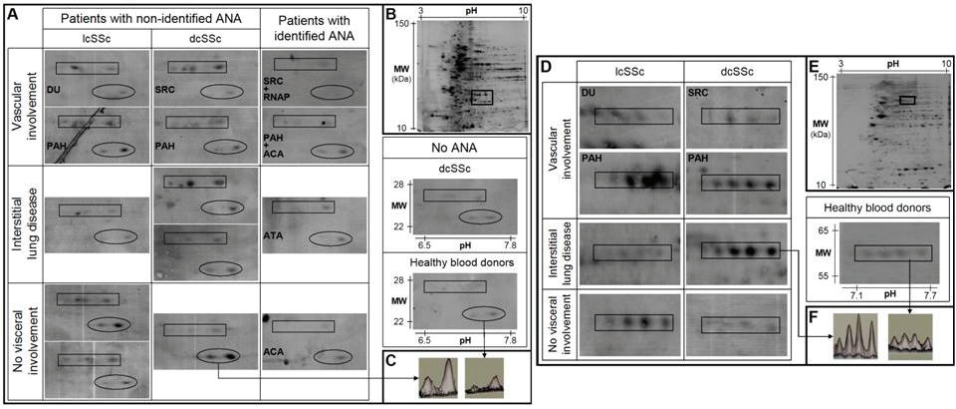
**IgG reactivities directed toward triosephosphate isomerase, superoxide dismutase mitochondrial precursor and heterogeneous nuclear ribonucleoprotein L**. **(A) **areas of 2-D membranes with IgG reactivities directed toward triosephosphate isomerase (rectangles) and superoxide dismutase mitochondrial precursor (ovals) in sera from patients with different subsets of SSc and from healthy blood donors in total protein extract. **(D) **Areas of 2-D membranes with IgG reactivities directed toward heterogeneous nuclear ribonucleoprotein L in sera from SSc patients with unidentified ANA and from healthy blood donors in enriched nuclear protein extract. 2-D silver-stained gel of total **(B) **and nuclear **(E) **protein extracts from HEp-2 cells. First dimension (*x*-axis): pH range from 3 to 10; second dimension: range from 150 to 10 kDa (*y*-axis). The areas delineated by rectangles in B (pH 6.5 to 7.8; 22 to 28 kDa) and D (pH 7.1 to 7.7; 55 to 65 kDa) correspond to the region of membranes magnified in A and D, respectively. **(C and F) **3-D representation of IgG reactivity peaks in a sera pool from three patients (left) and from the 12 healthy blood donors (right). ACA: anticentromere antibody; ANA: antinuclear antibody; ATA: antitopoisomerase I antibody; dcSSc: diffuse cutaneous systemic sclerosis; DU: digital ulcer; lcSSc: limited cutaneous systemic sclerosis; MW: molecular weight; PAH: pulmonary arterial hypertension; RNAP: anti-RNA polymerase III antibody; SRC: scleroderma renal crisis; SSc: systemic sclerosis.

We found 974 and 832 protein spots specifically stained by silver nitrate in HEp-2 cell total and enriched nuclear protein extracts, respectively (Figures 2B and 2E and Additional file 1). Major differences were observed between the two HEp-2 cell proteomes, corresponding to quantitative variation for a given protein spot as well as protein spots that were exclusively detected in one of the two protein extracts. In the total protein extract, a large number of protein spots stained with high intensity migrated between pH 4.0 and 7.0 and between 100 and 10 kDa. In the enriched nuclear protein extract, a lower number of protein spots was stained with high intensity and migrated between pH 5.0 and 9.0 and, with several exceptions, between 75 and 30 kDa.

After protein transfer and colloidal gold staining, we identified 658 ± 101 and 535 ± 66 protein spots on average per membrane in total and enriched nuclear protein extracts, respectively (data not shown). Again, quantitative and/or qualitative differences were observed between membranes transferred with one or the other of the protein extracts.

### IgG reactivities shared between SSc patients

In the 15 pools of sera from SSc patients, IgG recognised, on average per membrane, 142 ± 34 and 155 ± 47 protein spots in HEp-2 cell total and enriched nuclear protein extracts, respectively, with no significant difference between sera pools (data not shown). Overall, 43 and 33 protein spots were recognised by at least 75% of pools from patients with dcSSc and/or lcSSc in total and enriched nuclear protein extracts, respectively (Additional files 2 and 3). Thus, 14 and 4 proteins were identified by MS from the protein spots recognised by at least 75% of the 15 pools in total and enriched nuclear protein extracts, respectively (Table 2). A limited number of proteins were recognised by IgG from all pools of patients. All of these latter proteins were also recognised by IgG from HCs. Interestingly, α-enolase was recognised by IgG from all pools of patients in both extracts. Finally, among the spots recognised by IgG from the 10 pools of sera from patients with unidentified ANAs, 15 were specifically recognised by IgG from at least 4 of these 10 pools in total or enriched nuclear protein extracts (Table 3)

**Table 2:** HEp-2 cell proteins recognised by immunoglobulin G in at least 75% of sera pools from patients^a^

Protein	SwissProt accession number
Total protein extract	
Heat shock 70-kDa protein 1^b^	[SwissProt:HSP71_HUMAN]
Stress-induced phosphoprotein 1	[SwissProt:STIP1_HUMAN]
Protein disulfide-isomerase A3 precursor	[SwissProt:PDIA3_HUMAN]
Glial fibrillary acidic protein^b^	[SwissProt:GFAP_HUMAN]
α-enolase^b^	[SwissProt:ENOA_HUMAN]
Mannose-6 phosphate receptor-binding protein 1	[SwissProt:M6PBP_HUMAN]
40S ribosomal protein SA^b^	[SwissProt:RSSA_HUMAN]
Phosphoglycerate kinase 1	[SwissProt:PGK1_HUMAN]
Actin, cytoplasmic 1^b^	[SwissProt:ACTB_HUMAN]
Glyceraldehyde-3-phosphate dehydrogenase^b^	[SwissProt:G3P_HUMAN]
Heterogeneous nuclear ribonucleoproteins A2/B1	[SwissProt:ROA2_HUMAN]
Triosephosphate isomerase^b^	[SwissProt:TPIS_HUMAN]
Peroxiredoxin 6	[SwissProt:PRDX6_HUMAN]
Superoxide dismutase [Mn], mitochondrial precursor^b^	[SwissProt:SODM_HUMAN]
Enriched nuclear protein extract	
Heterogeneous nuclear ribonucleoprotein L^b^	[SwissProt:HNRPL_HUMAN]
Pre-mRNA processing factor 19	[SwissProt:PRP19_HUMAN]
α-enolase^b^	[SwissProt:ENOA_HUMAN]
Poly(rC)-binding protein 1	[SwissProt:PCBP1_HUMAN]

^a^SSc: systemic sclerosis. ^b^HEp-2 cell proteins recognised by all pools of sera from SSc patients with unidentified antinuclear antibodies.

**Table 3:** Proteins specifically recognised by IgG from at least four pools of patients with unidentified ANA

Protein ID on gel	HEp-2 cell protein	SwissProt accession number	MW th/es	pH_i _th/es	Number of unique identified peptides^#^	Total ion score	Best ion score	Sequence coverage (%)
550	Far upstream element-binding protein 2 (N)	[SwissProt:FUBP2_HUMAN]	73/80	6.8/7.1	10/17	554	108	37
553	Far upstream element-binding protein 2 (N)	[SwissProt:FUBP2_HUMAN]	73/79	6.8/7.3	11/17	864	153	32
554	Far upstream element-binding protein 2 (N)	[SwissProt:FUBP2_HUMAN]	73/79	6.8/7.5	10/17	598	105	34
617	Lamin A/C (N)	[SwissProt:LMNA_HUMAN]	74/73	6.6/7.0	11/29	680	127	50
762	RNA-binding protein FUS (N)	[SwissProt:FUS_HUMAN]	53/61	9.4/7.8	2/5	64	45	17
771	Ras GTPase-activating protein-binding protein 1 (N)	[SwissProt:G3BP1_HUMAN]	52/61	5.4/6.0	5/12	381	131	39
913	Lamin A/C (T)	[SwissProt:LMNA_HUMAN]	74/77	6.6/7.0	5/14	120	39	28
914	Lamin A/C (T)	[SwissProt:LMNA_HUMAN]	74/77	6.6/6.8	7/23	121	38	42
921	RuvB-like 1 (N)	[SwissProt:RUVB1_HUMAN]	50/50	6.0/6.8	8/16	591	131	50
	Protein DEK (N)	[SwissProt:DEK_HUMAN]	43/50	8.7/6.8	2/4	162	92	12
924	Heterogeneous nuclear ribonucleoprotein H (N)	[SwissProt:HNRH1_HUMAN]	49/49	5.9/6.4	8/15	440	80	53
1132	60-kDa heat shock protein, mitochondrial precursor (T)	[SwissProt:CH60_HUMAN]	61/61	5.7/5.5	7/15	176	36	28
1191	Serine/threonine protein phosphatase PP1-β catalytic subunit (N)	[SwissProt:PP1B_HUMAN]	37/34	5.8/6.1	2/10	62	41	35
1629	Annexin A1 (T)	[SwissProt:ANXA1_HUMAN]	39/38	6.6/6.7	6/13	233	73	50
2212	Stathmin (T)	[SwissProt:STMN1_HUMAN]	17/18	5.8/6.2	2/6	82	51	32
2039	Histone-binding protein RBBP4 (N)	[SwissProt:RBBP4_HUMAN]	48/48	4.7/5.1	7/10	414	103	27

^a^ANA: antinuclear antibody; FUS: fused in sarcoma; MW: molecular weight (in kilodaltons); N: proteins recognised in HEp-2 cell-enriched nuclear protein extract; pH_i_, intracellular pH; PP1: protein phosphatase 1; SSc: systemic sclerosis; T: proteins recognised in HEp-2 cell total protein extract; th/es: theoretical/estimated. ^b^Number of uniquely identified peptides in tandem mass spectrometry (MS/MS) and mass spectrometry + MS/MS searches.

### Comparison of IgG reactivities in sera from HCs and SSc patients

**Figure 3:**
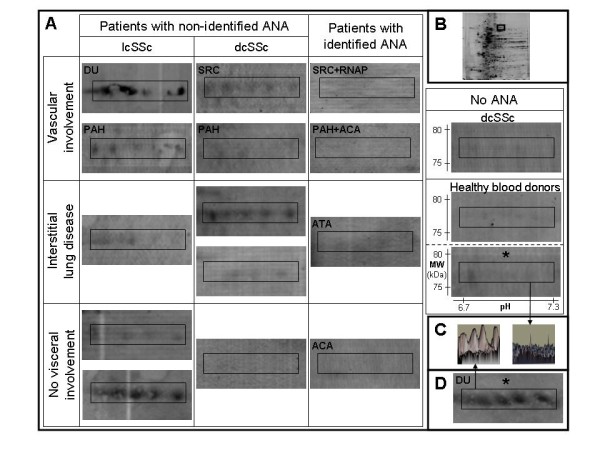
**IgG reactivities directed toward lamin A/C**. **(A) **Areas of 2-D membranes with IgG reactivities directed toward lamin A/C in sera from patients with different subsets of SSc and from healthy blood donors in total or nuclear (*) protein extracts from HEp-2 cells. **(B) **2-D silver-stained gel of HEp-2 cell total protein extract. The areas delineated by rectangles correspond to the region of membranes magnified in A (pH 6.7 to 7.3; 75 to 80 kDa). **(C) **3-D representation of IgG reactivity peaks in a sera pool from three patients (left) and from the 12 healthy blood donors (right). **(D) **IgG reactivities directed toward lamin A/C in enriched nuclear protein extract in the sera pool from patients with lcSSc, DU and unidentified ANA. ACA: anticentromere antibody; ANA: antinuclear antibody; ATA: antitopoisomerase I antibody; dcSSc: diffuse cutaneous systemic sclerosis; DU: digital ulcer; lcSSc: limited cutaneous systemic sclerosis; MW: molecular weight; PAH: pulmonary arterial hypertension; RNAP: anti-RNA polymerase III antibody; SRC: scleroderma renal crisis; SSc: systemic sclerosis.

Serum IgG from the pool of 12 HCs recognised 95 ± 1 and 108 ± 3 protein spots in total and enriched nuclear protein extracts, respectively. In the total protein extract, IgG reactivity for triosephosphate isomerase (TPI) and superoxide dismutase mitochondrial precursor (SOD2) was higher in the majority of pools of SSc patients, especially in those with sera from patients with unidentified ANAs, than in the pool of sera from HCs (Figure 2). Although IgG reactivity was slightly higher for SOD2 in sera from patients without visceral involvement, IgG reactivities did not differ between subgroups of patients for TPI or SOD2. In the enriched nuclear protein extract, IgG reactivity for heterogeneous nuclear ribonucleoprotein L (hnRNP L) was high in several sera pools from SSc patients with unidentified ANAs and low in the pool of sera from HCs (Figure 2). In both total and enriched nuclear protein extracts, IgG reactivity for lamin A/C was high in several sera pools from patients with unidentified ANAs (Figure 3). Interestingly, no IgG reactivity for lamin A/C was observed in sera pools from HCs and from patients with identified ANAs or without ANAs. Finally, IgG reactivity for lamin A/C was high in the pool of sera from patients with lcSSc, digital ulcers and unidentified ANAs in both total and enriched nuclear protein extracts (Figures 3A and 3D).

### Subset-specific IgG reactivities in sera from patients with unidentified ANAs

Using both groups of experiments performed with total and enriched nuclear protein extracts, we identified IgG reactivities that were specific for each phenotypic subset of patients with unidentified ANAs. MS identified a number of key target antigens (Table 4). Interestingly, with the exception of one subset, we identified at least one and up to four target antigens recognised by sera pools from each subset of patients with unidentified ANAs, including cofilin 1, peroxiredoxin 2 (PRDX2) and calreticulin (Table 4). One target antigen, eukaryotic translation initiation factor 5A-1, was identified in both the total and the enriched nuclear protein extracts from patients with the same disease subset.

**Table 4:** Proteins specifically recognised by IgG from patients with the same phenotype and expressing unidentified ANA^a^

Subset of patients	Protein ID on gel	HEp-2 cell protein	SwissProt accession number	MW th/es	pH_i _th/es	Number of unique identified peptides^#^	Total ion score	Best ion score	Sequence coverage (%)
dcSSc/SRC	1100	Calreticulin precursor (T)	[SwissProt:CALR_HUMAN]	48/63	4.3/4.4	5/16	136	36	25
	1420	Pre-mRNA splicing factor SPF27 (N)	[SwissProt:SPF27_HUMAN]	26/25	5.5/5.9	6/10	377	115	47
	1636	Eukaryotic translation initiation factor 5A-1 (N)	[SwissProt:IF5A1_HUMAN]	17/16	5.1/5.7	3/3	163	101	33
	2249	Eukaryotic translation initiation factor 5A-1 (T)	[SwissProt:IF5A1_HUMAN]	17/17	5.1/5.6	2/5	80	69	22
dcSSc/PAH	-	-	-	-	-				
dcSSc/ILD	589	Probable ATP-dependent RNA helicase DDX17 (N)	[SwissProt:DDX17_HUMAN]	72/76	8.8/8.0	8/20	207	35	36
	1101	Poly(rC)-binding protein 2 (N)	[SwissProt:PCBP2_HUMAN]	39/39	6.3/6.9	5/10	132	56	41
	1151	Serine/threonine protein phosphatase PP1-α catalytic subunit (N)	[SwissProt:PP1A_HUMAN]	37/35	5.9/6.5	10/17	476	114	61
dcSSc*	1417	DNA-directed RNA polymerases I, II and III, subunit RPABC1 (N)	[SwissProt:RPAB1_HUMAN]	25/25	5.7/6.3	2/4	150	117	21
	2163	Cofilin 1 (T)	[SwissProt:COF1_HUMAN]	19/19	8.2/9.5	3/7	134	72	54
lcSSc/DU	2317	Histone H2A type 1-J (T)	[SwissProt:H2A1J_HUMAN]	14/16	10.9/6.1	2/3	37	20	27
lcSSc/PAH	882	Telomeric repeat binding factor 2-interacting protein 1 (N)	[SwissProt:TE2IP_HUMAN]	44/52	4.6/4.9	9/15	286	71	48
	1119	Heterogeneous nuclear ribonucleoprotein A/B (N)	[SwissProt:ROAA_HUMAN]	36/38	8.2/6.5	3/5	55	27	15
	2079	Peroxiredoxin 2 (T)	[SwissProt:PRDX2_HUMAN]	22/23	5.7/6.0	5/7	143	40	26
lcSSc/ILD	901	78-kDa glucose-regulated protein precursor (T)	[SwissProt:GRP78_HUMAN]	72/76	5.1/5.4	13/29	711	121	28
	2063	ATP-dependent DNA helicase 2, subunit 1 (N)	[SwissProt:KU70_HUMAN]	70/70	6.2/6.9	3/14	89	45	29
lcSSc*	820	U4/U6 small nuclear ribonucleoprotein Prp31 (N)	[SwissProt:PRP31_HUMAN]	55/57	5.6/6.4	3/7	112	64	16
	1478	Calumenin precursor (T)	[SwissProt:CALU_HUMAN]	37/44	4.5/4.6	3/7	82	39	29
	1895	Tumour protein D54 (T)	[SwissProt:TPD54_HUMAN]	22/29	5.3/5.6	1/3	47	47	23

^a^ANA: antinuclear antibody; dcSSc: diffuse cutaneous systemic sclerosis; DU: digital ulcer; ILD: interstitial lung disease; lcSSc: limited cutaneous systemic sclerosis; MW: molecular weight (in kilodaltons); N: proteins recognised in HEp-2 cell-enriched nuclear protein extract; PAH: pulmonary arterial hypertension; SRC: scleroderma renal crisis; SSc: systemic sclerosis; T: proteins recognised in HEp-2 cell total protein extract; th/es: theoretical/estimated. ^b^Number of unique identified peptides in MS/MS and in MS+MS/MS searches. ^c^Without visceral involvement.

### Biological network analysis of identified autoantibody specificities

**Figure 4:**
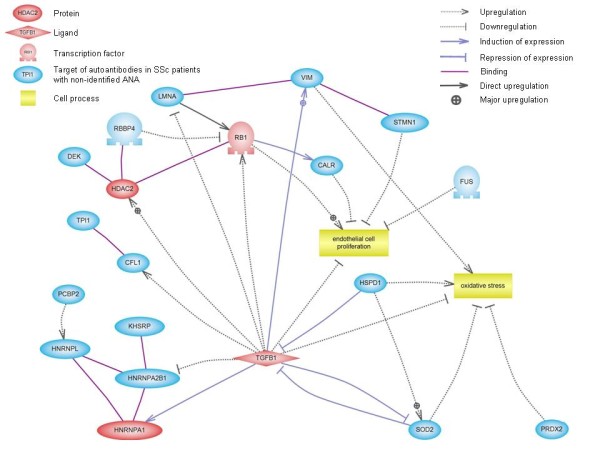
**Signalling network of proteins identified as major targets of autoantibodies in patients with unidentified ANA**. This schematic representation, created by using Pathway Studio software, shows the connectivity between TGF-β and HEp-2 cell proteins identified as major targets of autoantibodies in SSc patients with unidentified ANA. Protein entities belonging to different functional groups are represented as different shapes. ANA: antinuclear antibody; CALR: calreticulin; CFL1: cofilin 1; FUS: fused in sarcoma; HDAC2: histone deacetylase 2; HNRNPA1: heterogeneous nuclear ribonucleoprotein A1; HNRNPA2B1: heterogeneous nuclear ribonucleoprotein A2/B1; HNRNPL: heterogeneous nuclear ribonucleoprotein L; HSPD1: heat shock 60-kDa protein 1; KHSRP: KH-type splicing regulatory protein (far upstream element-binding protein 2); LMNA: lamin A/C; PCBP2: poly(rC)-binding protein 2; PRDX2: peroxiredoxin 2; RB1: retinoblastoma-associated protein; RBBP4: retinoblastoma-binding protein 4; SOD2: superoxide dismutase 2, mitochondrial; SSc: systemic sclerosis; STMN1: stathmin 1; TGFB1: transforming growth factor β1; TPI1: triosephosphate isomerase 1; VIM: vimentin.

Lists of HEp-2 cell proteins specifically recognised and/or recognised with high intensity by IgG from SSc patients were analysed by using Pathway Studio software. Interestingly, most of these proteins were involved in the transforming growth factor β (TGF-β) pathway (Additional file 4). From this network, we wanted to focus on molecules recognised by IgG from SSc patients with unidentified ANAs. This allowed us to depict the signalling network between TGF-β and HEp-2 cell proteins identified as major targets of autoantibodies in SSc patients with unidentified ANAs (Figure 4). Thus, the expression of these proteins can be either increased or decreased by TGF-β. Interestingly, some of these proteins are involved in the pathophysiological process of SSc.

## Discussion

In the present work, we have identified a number of new target antigens for autoantibodies in SSc patients that are either shared among patients or specific to a given phenotype. For some antigens, including TPI, SOD2, hnRNP L and lamin A/C, IgG reactivity was higher in sera pools from patients than in pools from HCs. TPI, a glycolytic enzyme localised in the cytoplasm, is one of the nine proteins specifically identified in whole saliva from patients with dcSSc as compared with HCs [17]. Interestingly, we recently identified another glycolytic enzyme, α-enolase, as a target of antifibroblast antibodies in SSc patients, particularly those with ILD and/or ATAs [18,19]. SOD2 is a mitochondrial metalloenzyme that catalyses the dismutation of the superoxide anion to hydrogen peroxide and oxygen and protects against reactive oxygen species (ROS). Thus, autoantibodies directed against SOD2 might impair the enzyme function and favour ROS accumulation. This finding could be relevant to the pathogenesis of SSc, because a major increase in ROS level is a hallmark of SSc [20]. Interestingly, Dalpke *et al*. [21] reported that a hyperimmune serum against SOD2 inhibited the protective effects of SOD2 on endothelial cells exposed to oxidative stress. In addition, downregulation of SOD2 expression was described in osteoarthritis [22], and anti-TPI antibodies have been identified in several autoimmune conditions, including neuropsychiatric systemic lupus erythematosus (SLE) [23], and in osteoarthritis [24].

Lamins A and C are both encoded by the *LMNA *gene and represent major constituents of the inner nuclear membrane. Mutations of this gene have been identified in a number of conditions, including Hutchinson-Gilford progeria syndrome [25], which represents a major differential diagnosis of juvenile SSc. The most frequent mutation responsible for progeria creates a truncated progeria mutant lamin A (progerin), which accumulates within the nuclei of human vascular cells and may be directly responsible for vascular involvement in progeria [26]. The identification of lamin as a major target of autoantibodies in SSc patients precludes the potential role of modified and/or dysfunctional lamin and/or antilamin autoantibodies in the pathogenesis of SSc. Antilamin antibodies were found in sera from patients with SLE [27] and antiphospholipid syndrome [28] as well as in a patient with linear morphea [29].

HnRNP L is a nuclear protein associated with hnRNP complexes and takes part in the processing of pre-mRNA. Anti-hnRNP L antibodies were identified in a small cohort of SSc patients in association with anti-hnRNP A/B antibodies [30]. HnRNP L was also identified as a target of autoantibodies in New Zealand White × BXSB mice with SLE and antiphospholipid syndrome [31].

Our analysis revealed that PRDX2, cofilin 1 and calreticulin were specifically recognised by IgG from phenotypic subsets of patients with unidentified ANAs. Other target antigens listed in Table 4 might also be relevant and should be tested in further work. PRDX2 is a peroxidase that eliminates endogenous ROS produced in response to growth factors such as platelet-derived growth factor (PDGF). PRDX2 influences oxidative and heat stress resistance [32] and inhibits PDGF signalling and vascular remodelling [33]. Interestingly, PRDX2 has recently been identified as a target of anti-endothelial cell antibodies in systemic vasculitis [34].

Cofilin 1 is a regulator of actin depolymerisation. Cofilin is a major effector of nicotinamide adenine dinucleotide phosphate (NADPH) oxidase 1-mediated migration, and NADPH oxidase 1 plays a critical role in neointima formation by mediating vascular smooth muscle cell migration, proliferation and extracellular matrix production [35]. Moreover, regulation of the phosphorylation state of cofilin controls PDGF-induced migration of human aortic smooth muscle cells [36]. Anti-cofilin 1 antibodies have been detected in a few patients with rheumatoid arthritis, SLE or polymyositis and/or dermatomyositis [37].

Calreticulin is an endoplasmic reticulum chaperone and an intracellular calcium-binding protein and thus is involved in signal transduction pathways. In apoptotic cells, calreticulin is translocated to the cell surface, conferring immunogenicity of cell death [38]. Calreticulin has been described as a potential cell surface receptor involved in cell penetration of anti-DNA antibodies in patients with SLE [39]. Anticalreticulin antibodies have been reported in patients with celiac disease and SLE [40,41].

Interestingly, we determined that several autoantigens recognised by IgG from SSc patients were involved in the TGF-β pathway. In the pathophysiology of SSc, fibroblast proliferation and accumulation of extracellular matrix result from uncontrolled activation of the TGF-β pathway and from excess synthesis of connective tissue growth factor, PDGF, proinflammatory cytokines and ROS [3]. Thus, increased expression and/or modified structure or fragmentation in the presence of ROS of a number of proteins involved in the TGF-β pathway could trigger specific immune responses in these patients. Casciola-Rosen *et al*. [42] reported on the sensitivity of scleroderma antigens to ROS-induced fragmentation in this setting, possibly through ischemia-reperfusion injury as the potential initiator of the autoimmune process in SSc.

The combined use of 2-DE and immunoblotting offers an interesting approach to identifying target antigens of autoantibodies [10,13]. We used HEp-2 cells as sources of autoantigens because these cells are routinely used to detect ANAs. Although not directly relevant to the pathogenesis of SSc, we thought it more appropriate to use these cells as sources of autoantigens because we were looking for additional targets to ANAs. Additional validation studies with sera from patients with other connective tissue diseases are necessary. In addition, 2-DE and immunoblotting were not adapted to test a large number of sera, and thus further experiments using ELISA with recombinant proteins are necessary, which will allow for validation of the target antigens and screening of a large number of patients.

However, our work has several additional limitations. Less than 1,000 protein spots were stained in the reference gel of the total protein extract. Therefore, a number of proteins were probably lost at each step of the technique, depending on their charge, molecular weight, subcellular localisation and/or abundance in the cell. Topoisomerase II is not detected by traditional methods of 2-DE [43], and we failed to identify topoisomerase I or centromeric protein B as target antigens of IgG autoantibodies, whereas these antigens are easily detected in 1-D gels [6,44,45]. Anti-topoisomerase I and anti-RNA polymerase III antibodies preferentially recognise a discontinuous or conformational epitope that may not be detected in 2-D gels [46,47]. As expected, none of the identified antigens was located at the cell surface, since protein extraction for 2-DE does not allow the identification of membrane proteins.

## Conclusions

We have identified new target autoantigens in SSc patients, a number of which are involved in the TGF-β pathway. Although these data must be confirmed by other groups and in large cohorts of patients with SSc or other connective tissue diseases, these new autoantibody specificities could represent major advances in the diagnosis and prognosis of patients with SSc.

## Abbreviations

2-DE: two-dimensional electrophoresis; ACA: anti-centromere antibody; ANA: antinuclear antibodies; ATA: anti-topoisomerase I antibody; dcSSc: diffuse cutaneous systemic sclerosis; EDTA: ethylenediaminetetraacetic acid; EGTA: ethyleneglycoltetraacetic acid; HC: healthy controls; hnRNP L: heterogeneous nuclear ribonucleoprotein L; ILD: interstitial lung disease; lcSSc: limited cutaneous systemic sclerosis; MALDI: matrix-assisted laser desorption/ionization; MS: mass spectrometry; MS/MS: tandem mass spectrometry; PAH: pulmonary arterial hypertension; PDGF: platelet-derived growth factor; PRDX2: peroxiredoxin 2; PVDF: polyvinylidene difluoride; ROS: reactive oxygen species; SLE: systemic lupus erythematosus; SOD2: superoxide dismutase mitochondrial precursor; SRC: scleroderma renal crisis; SSc: systemic sclerosis; TGF: transforming growth factor; TOF: time of flight; TPI: triosephosphate isomerase.

## Competing interests

The authors declare that they have no competing interests.

## Authors' contributions

GB participated in study design, performed most of the experiments and drafted the manuscript. HD contributed to the experiments and revised the manuscript. MCT contributed to the study design and the interpretation of data and revised the manuscript. CB and LC performed mass spectrometry experiments and revised the manuscript. CF performed Pathway Studio analysis and revised the manuscript. GW supervised the recruitment of healthy blood donors and revised the manuscript. LG supervised the recruitment of patients with systemic sclerosis and revised the manuscript. LM directed the study design, supervised the recruitment of patients with systemic sclerosis, contributed to the interpretation of data and drafted the manuscript. All authors read and approved the final manuscript.

## Supplementary Material

Additional file 1 **Supplemental Figure S1. HEp-2 cell proteomes. (A) **2-D silver-stained gel of total protein extract and **(C) **enriched nuclear protein extract. First dimension (*x*-axis): pH range 3 to 10; second dimension: range from 150 to 10 kDa (*y*-axis). B and D are magnifications of the delineated zones in A and C, respectively. Proteins of interest are indicated by the protein ID provided by ImageMaster 2D Platinum 6.0 software or their SwissProt accession numbers (see Tables 2, 3 and 4 for the names of these proteins). Protein spots delineated by rectangles are different isoforms of the same protein. Click here for file

Additional file 2 **Supplemental Table S1**. Proteins recognised by immunoglobulin G (IgG) in at least 75% of pools of patients with diffuse cutaneous systemic sclerosis (dcSSc) and/or limited cutaneous systemic sclerosis (lcSSc) in HEp-2 cell total protein extract. Click here for file

Additional file 3 **Supplemental Table S2**. Proteins recognised by immunoglobulin G in at least 75% of pools of patients with dcSSc and/or lcSSc in HEp-2 cell-enriched nuclear protein extract. Click here for file

Additional file 4 **Supplemental Figure S2. Signalling network of HEp-2 cell proteins specifically recognised and/or recognised with high intensity by IgG from SSc patients**. This schematic representation, created by using Pathway Studio software, shows the connectivity between IgG target antigens and TGF-β. Protein entities belonging to different functional groups are represented as different shapes. CALR: calreticulin; CFL1: cofilin 1; DEK: protein DEK; ENO1: enolase 1α; FUS: fused in sarcoma; HDAC1: histone deacetylase 1; HDAC2: histone deacetylase 2; HNRNPA1: heterogeneous nuclear ribonucleoprotein A1; HNRNPA2B1: heterogeneous nuclear ribonucleoprotein A2/B1; HNRNPH1: heterogeneous nuclear ribonucleoprotein H1; HNRNPK: heterogeneous nuclear ribonucleoprotein K; HNRNPL: heterogeneous nuclear ribonucleoprotein L; HSPD1: heat shock 60-kDa protein 1; KHSRP: KH-type splicing regulatory protein (far upstream element-binding protein 2); LMNA: lamin A/C; POLR2A: polymerase (RNA) II (DNA-directed) polypeptide A; POLR2E: polymerase (RNA) II (DNA-directed) polypeptide E; PRDX2: peroxiredoxin 2; RBBP4: retinoblastoma-binding protein 4; RUVBL1: RuvB-like 1; SOD2: superoxide dismutase 2, mitochondrial; SSc: systemic sclerosis; STMN1: stathmin 1; TBP: TATA box-binding protein; TGFB1: transforming growth factor β1; TOP1: topoisomerase (DNA) I; TPI1: triosephosphate isomerase 1; VIM: vimentin. Click here for file
