# Supplementary figures and images for: Identification of new autoantibody specificities directed at proteins involved in the transforming growth factor β pathway in patients with systemic sclerosis
Source: Arthritis Res Ther. 2011 May 13;13(3):R74. doi: 10.1186/ar3336 (PMC3218884; doi:10.1186/ar3336)

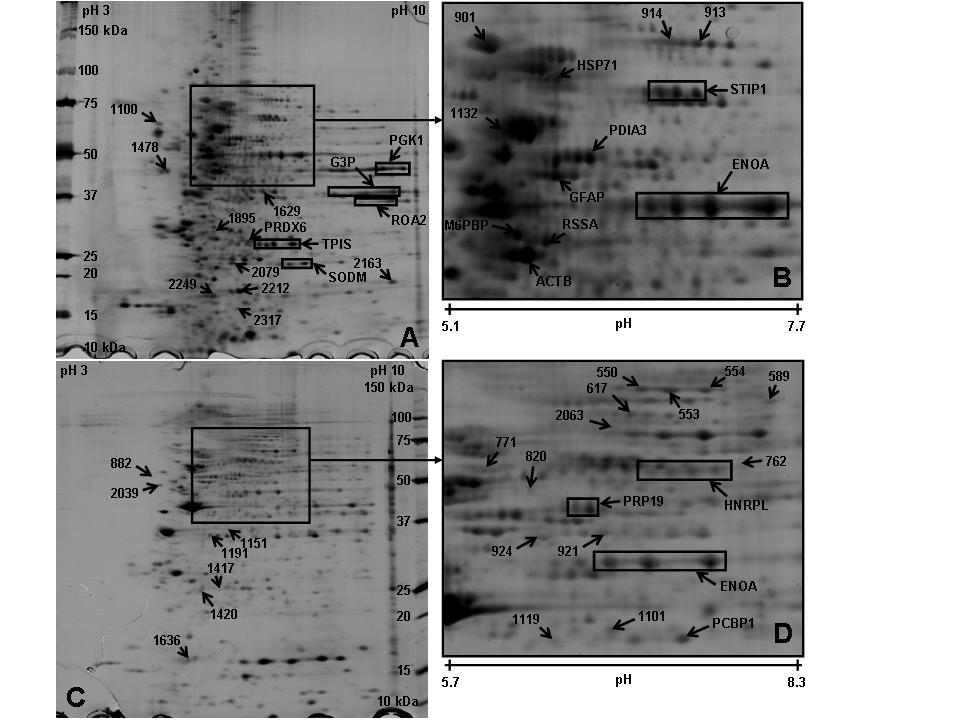

Supplement: Additional file 1 — Supplemental Figure S1. HEp-2 cell proteomes. (A) 2-D silver-stained gel of total protein extract and (C) enriched nuclear protein extract. First dimension (x-axis): pH range 3 to 10; second dimension: range from 150 to 10 kDa (y-axis). B and D are magnifications of the delineated zones in A and C, respectively. Proteins of interest are indicated by the protein ID provided by ImageMaster 2D Platinum 6.0 software or their SwissProt accession numbers (see Tables 2, 3 and 4 for the names of these proteins). Protein spots delineated by rectangles are different isoforms of the same protein. [file ar3336-S1.JPEG]

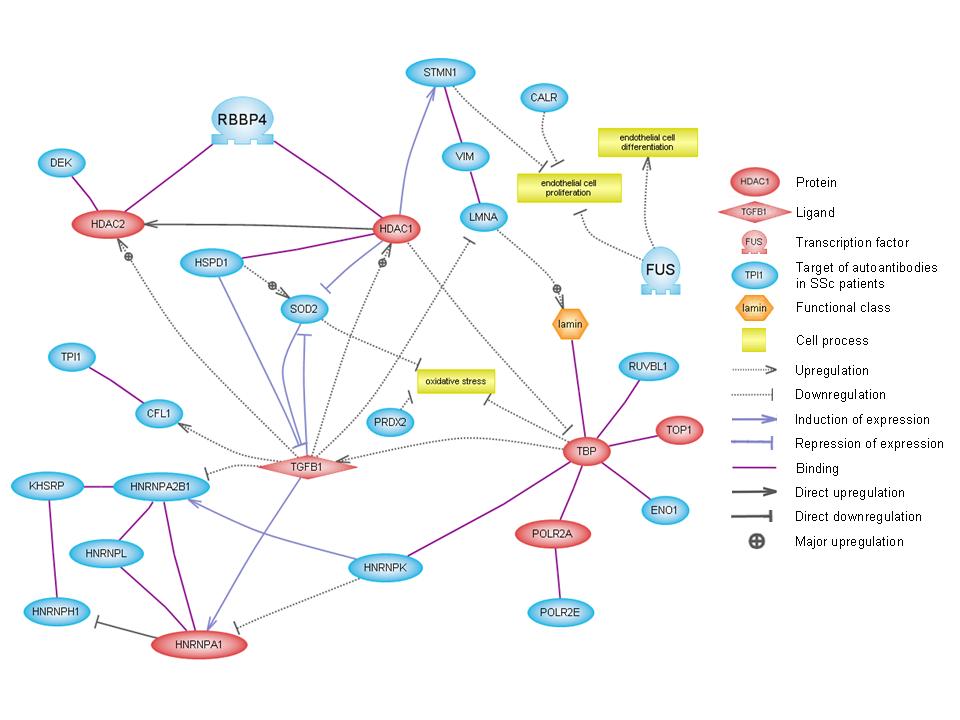

Supplement: Additional file 4 — Supplemental Figure S2. Signalling network of HEp-2 cell proteins specifically recognised and/or recognised with high intensity by IgG from SSc patients. This schematic representation, created by using Pathway Studio software, shows the connectivity between IgG target antigens and TGF-β. Protein entities belonging to different functional groups are represented as different shapes. CALR: calreticulin; CFL1: cofilin 1; DEK: protein DEK; ENO1: enolase 1α; FUS: fused in sarcoma; HDAC1: histone deacetylase 1; HDAC2: histone deacetylase 2; HNRNPA1: heterogeneous nuclear ribonucleoprotein A1; HNRNPA2B1: heterogeneous nuclear ribonucleoprotein A2/B1; HNRNPH1: heterogeneous nuclear ribonucleoprotein H1; HNRNPK: heterogeneous nuclear ribonucleoprotein K; HNRNPL: heterogeneous nuclear ribonucleoprotein L; HSPD1: heat shock 60-kDa protein 1; KHSRP: KH-type splicing regulatory protein (far upstream element-binding protein 2); LMNA: lamin A/C; POLR2A: polymerase (RNA) II (DNA-directed) polypeptide A; POLR2E: polymerase (RNA) II (DNA-directed) polypeptide E; PRDX2: peroxiredoxin 2; RBBP4: retinoblastoma-binding protein 4; RUVBL1: RuvB-like 1; SOD2: superoxide dismutase 2, mitochondrial; SSc: systemic sclerosis; STMN1: stathmin 1; TBP: TATA box-binding protein; TGFB1: transforming growth factor β1; TOP1: topoisomerase (DNA) I; TPI1: triosephosphate isomerase 1; VIM: vimentin. [file ar3336-S4.JPEG]
