# Supplementary material for: Identification of new autoantibody specificities directed at proteins involved in the transforming growth factor β pathway in patients with systemic sclerosis
Source: Arthritis Res Ther. 2011 May 13;13(3):R74. doi: 10.1186/ar3336 (PMC3218884; doi:10.1186/ar3336)
Supplement: Additional file 2 — Supplemental Table S1. Proteins recognised by immunoglobulin G (IgG) in at least 75% of pools of patients with diffuse cutaneous systemic sclerosis (dcSSc) and/or limited cutaneous systemic sclerosis (lcSSc) in HEp-2 cell total protein extract. [file ar3336-S2.DOC]

**Supplemental Table 1.** Proteins recognised by immunoglobulin G in at least 75% of pools of patients with dcSSc and/or lcSSc in HEp-2 cell total protein extract.

| Protein ID  on gel | Protein | SwissProt accession  number | MW  th/es | pHi  th/es | dcSSc | lcSSc | Number  of unique  identified  peptides^#^ | Total  ion  score | Best  ion  score | Sequence  coverage  (%) |
| --- | --- | --- | --- | --- | --- | --- | --- | --- | --- | --- |
| 817 | Heat shock protein HSP 90-beta | [SwissProt:HS90B_HUMAN] | 84/90 | 5.0/5.5 |  | x | 3/15 | 46 | 18 | 13 |
| 913 | Lamin-A/C | [SwissProt:LMNA_HUMAN] | 74/77 | 6.6/7.0 |  | x | 5/14 | 120 | 39 | 28 |
| 991 | Heat shock cognate 71 kDa protein | [SwissProt:HSP7C_HUMAN] | 71/71 | 5.4/5.8 |  | x | 9/20 | 346 | 83 | 41 |
| 1016 | Heat shock 70 kDa protein 1 | [SwissProt:HSP71_HUMAN] | 70/69 | 5.5/6.0 | x | x | 10/25 | 218 | 59 | 25 |
| 1043 | Stress-induced-phosphoprotein 1 | [SwissProt:STIP1_HUMAN] | 63/67 | 6.4/6.8 | x | x | 2/12 | 48 | 27 | 32 |
| 1046 | Lamin-A/C | [SwissProt:LMNA_HUMAN] | 74/67 | 6.6/6.7 | x |  | 4/22 | 133 | 27 | 22 |
|  | Stress-induced-phosphoprotein 1 | [SwissProt:STIP1_HUMAN] | 63/67 | 6.4/6.7 | x |  | 4/14 | 123 | 55 | 28 |
| 1052 | Stress-induced-phosphoprotein 1 | [SwissProt:STIP1_HUMAN] | 63/67 | 6.4/7.0 | x | x | 3/15 | 56 | 28 | 36 |
| 1078 | Lamin-A/C | [SwissProt:LMNA_HUMAN] | 74/65 | 6.6/6.9 |  | x | 9/21 | 276 | 53 | 32 |
| 1124 | 60 kDa heat shock protein, mitochondrial precursor | [SwissProt:CH60_HUMAN] | 61/62 | 5.7/5.7 | x |  | 12/22 | 502 | 113 | 56 |
| 1126 | Heterogeneous nuclear ribonucleoprotein K | [SwissProt:HNRPK_HUMAN] | 51/62 | 5.4/5.9 |  | x | 5/18 | 131 | 41 | 22 |
| 1195 | Vimentin | [SwissProt:VIME_HUMAN] | 54/56 | 5.1/5.6 | x |  | 9/26 | 236 | 57 | 32 |
| 1198 | Succinyl-CoA:3-ketoacid-coenzyme A transferase 1, mitochondrial precursor | [SwissProt:SCOT_HUMAN] | 57/57 | 7.1/6.7 |  | x | 6/11 | 160 | 69 | 32 |
| 1204 | Protein disulfide-isomerase A3 precursor | [SwissProt:PDIA3_HUMAN] | 57/56 | 6.0/6.2 | x | x | 10/21 | 314 | 49 | 32 |
| 1230 | T-complex protein 1 subunit beta | [SwissProt:TCPB_HUMAN] | 58/55 | 6.0/6.6 |  | x | 2/18 | 141 | 14 | 30 |
| 1258 | Heterogeneous nuclear ribonucleoprotein H | [SwissProt:HNRH1_HUMAN] | 49/53 | 5.9/6.1 | x | x | 1/9 | 72 | 72 | 30 |
|  | Glial fibrillary acidic protein, astrocyte | [SwissProt:GFAP_HUMAN] | 50/53 | 5.4/6.1 | x | x | 1/7 | 50 | 50 | 18 |
| 1278 | Tubulin beta chain | [SwissProt:TBB5_HUMAN] | 50/53 | 4.8/5.4 |  | x | 7/14 | 135 | 42 | 44 |
| 1279 | Glial fibrillary acidic protein, astrocyte | [SwissProt:GFAP_HUMAN] | 50/53 | 5.4/5.9 | x | x | 1/4 | 56 | 56 | 11 |
| 1320 | Alpha-enolase | [SwissProt:ENOA_HUMAN] | 47/49 | 7.0/6.8 | x | x | 7/20 | 371 | 84 | 64 |
| 1321 | Alpha-enolase | [SwissProt:ENOA_HUMAN] | 47/49 | 7.0/7.1 | x | x | 9/17 | 424 | 131 | 58 |
| 1327 | Alpha-enolase | [SwissProt:ENOA_HUMAN] | 47/49 | 7.0/6.6 | x | x | 3/17 | 106 | 47 | 56 |
| 1328 | Alpha-enolase | [SwissProt:ENOA_HUMAN] | 47/49 | 7.0/7.5 | x | x | 7/19 | 324 | 79 | 61 |
| 1356 | Alpha-enolase | [SwissProt:ENOA_HUMAN] | 47/49 | 7.0/8.2 | x | x | 2/17 | 53 | 27 | 20 |
| 1365 | KRT17 protein | [SwissProt:K1C17_HUMAN] | 41/49 | 4.9/5.4 | x |  | 6/21 | 158 | 37 | 46 |
| 1410 | HNRPF protein | [SwissProt:HNRPF_HUMAN] | 46/47 | 5.4/5.8 |  | x | 5/10 | 187 | 69 | 32 |
| 1416 | Mannose-6-phosphate receptor-binding protein 1 | [SwissProt:M6PBP_HUMAN] | 47/46 | 5.3/5.7 | x |  | 4/8 | 179 | 58 | 25 |
| 1432 | 40S ribosomal protein SA | [SwissProt:RSSA_HUMAN] | 33/45 | 4.8/5.9 | x | x | 1/5 | 45 | 45 | 25 |
| 1448 | Phosphoglycerate kinase 1 | [SwissProt:PGK1_HUMAN] | 45/45 | 8.3/9.3 | x | x | 6/19 | 118 | 39 | 26 |
| 1450 | Phosphoglycerate kinase 1 | [SwissProt:PGK1_HUMAN] | 45/45 | 8.3/9.7 | x |  | 2/7 | 83 | 66 | 29 |
| 1472 | Actin, cytoplasmic 1 | [SwissProt:ACTB_HUMAN] | 42/44 | 5.3/5.7 | x | x | 6/10 | 290 | 72 | 35 |
| 1627 | Glyceraldehyde-3-phosphate dehydrogenase | [SwissProt:G3P_HUMAN] | 36/38 | 8.6/9.5 | x | x | 2/5 | 75 | 48 | 17 |
| 1645 | Glyceraldehyde-3-phosphate dehydrogenase | [SwissProt:G3P_HUMAN] | 36/38 | 8.6/8.9 | x | x | 2/6 | 142 | 60 | 28 |
| 1653 | Glyceraldehyde-3-phosphate dehydrogenase | [SwissProt:G3P_HUMAN] | 36/38 | 8.6/8.2 |  | x | 3/8 | 195 | 81 | 34 |
| 1681 | Heterogeneous nuclear ribonucleoprotein D-like | [SwissProt:HNRDL_HUMAN] | 47/36 | 9.6/6.4 |  | x | 3/5 | 97 | 35 | 15 |
| 1703 | Heterogeneous nuclear ribonucleoproteins A2/B1 | [SwissProt:ROA2_HUMAN] | 37/36 | 9.0/9.4 | x | x | 3/6 | 72 | 25 | 20 |
| 1722 | Heterogeneous nuclear ribonucleoproteins A2/B1 | [SwissProt:ROA2_HUMAN] | 37/35 | 9.0/8.8 | x |  | 5/14 | 124 | 48 | 28 |
| 1733 | Elongation factor 1-delta | [SwissProt:EF1D_HUMAN] | 31/35 | 4.9/5.4 | x |  | 3/10 | 91 | 59 | 34 |
| 1960 | Triosephosphate isomerase | [SwissProt:TPIS_HUMAN] | 27/27 | 6.5/6.9 | x |  | 10/18 | 522 | 77 | 83 |
| 1965 | Triosephosphate isomerase | [SwissProt:TPIS_HUMAN] | 27/27 | 6.5/7.3 | x | x | 8/15 | 339 | 82 | 75 |
| 1967 | Peroxiredoxin-6 | [SwissProt:PRDX6_HUMAN] | 25/27 | 6.0/6.8 |  | x | 8/11 | 316 | 53 | 87 |
| 1969 | Peroxiredoxin-4 | [SwissProt:PRDX4_HUMAN] | 31/27 | 5.9/6.1 | x |  | 3/6 | 132 | 62 | 31 |
| 1975 | Proteasome subunit alpha type-5 | [SwissProt:PSA5_HUMAN] | 27/27 | 4.7/5.0 |  | x | 6/8 | 211 | 64 | 54 |
|  | Membrane-associated progesterone receptor component 2 | [SwissProt:PGRC2_HUMAN] | 24/27 | 4.8/5.0 |  | x | 3/5 | 66 | 31 | 34 |
| 2068 | Prohibitin | [SwissProt:PHB_HUMAN] | 30/24 | 5.6/5.2 | x |  | 2/3 | 67 | 39 | 12 |
| 2077 | Superoxide dismutase [Mn], mitochondrial precursor | [SwissProt:SODM_HUMAN] | 22/23 | 6.9/7.6 | x | x | 5/8 | 227 | 71 | 53 |

^#^ Number of unique identified peptides in MS/MS and in MS+MS/MS searches. dcSSc: diffuse cutaneous SSc; lcSSc limited cutaneous SSc; MW: molecular weight (kDa); SSc: systemic sclerosis; th/es: theoretical/estimated.
