# Supplementary material for: Identification of new autoantibody specificities directed at proteins involved in the transforming growth factor β pathway in patients with systemic sclerosis
Source: Arthritis Res Ther. 2011 May 13;13(3):R74. doi: 10.1186/ar3336 (PMC3218884; doi:10.1186/ar3336)
Supplement: Additional file 3 — Supplemental Table S2. Proteins recognised by immunoglobulin G in at least 75% of pools of patients with dcSSc and/or lcSSc in HEp-2 cell-enriched nuclear protein extract. [file ar3336-S3.DOC]

**Supplemental Table 2.** Proteins recognised by immunoglobulin G in at least 75% of pools of patients with dcSSc and/or lcSSc in HEp-2 cell nuclear protein extract.

| Protein ID  on gel | Protein | SwissProt accession  number | MW  th/es | pHi  th/es | dcSSc | lcSSc | Number  of unique  identified  peptides^#^ | Total  ion  score | Best  ion  score | Sequence  coverage  (%) |
| --- | --- | --- | --- | --- | --- | --- | --- | --- | --- | --- |
| 553 | Far upstream element-binding protein 2 | [SwissProt:FUBP2_HUMAN] | 73/79 | 6.8/7.3 |  | x | 11/17 | 864 | 153 | 32 |
| 554 | Far upstream element-binding protein 2 | [SwissProt:FUBP2_HUMAN] | 73/79 | 6.8/7.5 | x |  | 10/17 | 598 | 105 | 34 |
| 617 | Lamin-A/C | [SwissProt:LMNA_HUMAN] | 74/73 | 6.6/7.0 |  | x | 10/17 | 598 | 105 | 34 |
| 691 | Heat shock cognate 71 kDa protein | [SwissProt:HSP7C_HUMAN] | 71/67 | 5.4/5.9 | x |  | 2/6 | 78 | 41 | 10 |
| 747 | Paraspeckle component 1 | [SwissProt:PSPC1_HUMAN] | 59/62 | 6.3/6.8 | x |  | 3/10 | 110 | 54 | 18 |
| 760 | Lamin-A/C | [SwissProt:LMNA_HUMAN] | 74/61 | 6.6/7.1 | x |  | 11/25 | 374 | 60 | 38 |
| 767 | Heterogeneous nuclear ribonucleoprotein L | [SwissProt:HNRPL_HUMAN] | 60/61 | 6.7/7.6 | x | x | 6/14 | 152 | 50 | 41 |
| 771 | Ras GTPase-activating protein-binding protein 1 | [SwissProt:G3BP1_HUMAN] | 52/61 | 5.4/6.0 | x |  | 5/12 | 381 | 131 | 39 |
| 772 | Heterogeneous nuclear ribonucleoprotein L | [SwissProt:HNRPL_HUMAN] | 60/60 | 6.7/7.5 | x | x | 6/15 | 143 | 55 | 42 |
| 778 | Heterogeneous nuclear ribonucleoprotein L | [SwissProt:HNRPL_HUMAN] | 60/60 | 6.7/7.3 |  | x | 6/16 | 118 | 31 | 42 |
| 795 | Pyruvate kinase isozymes M1/M2 | [SwissProt:KPYM_HUMAN] | 58/58 | 8.0/9.2 | x |  | 6/15 | 196 | 43 | 39 |
| 796 | Heterogeneous nuclear ribonucleoprotein K | [SwissProt:HNRPK_HUMAN] | 51/58 | 5.4/6.0 | x |  | 6/17 | 363 | 77 | 48 |
| 809 | U4/U6 small nuclear ribonucleoprotein Prp4 | [SwissProt:PRP4_HUMAN] | 58/58 | 7.1/8.0 | x |  | 5/11 | 172 | 57 | 27 |
|  | Pyruvate kinase isozymes M1/M2 | [SwissProt:KPYM_HUMAN] | 58/58 | 8.0/8.0 | x |  | 4/12 | 129 | 61 | 30 |
| 857 | Pre-mRNA-processing factor 19 | [SwissProt:PRP19_HUMAN] | 55/53 | 6.1/6.7 | x |  | 4/11 | 150 | 63 | 44 |
| 860 | Pre-mRNA-processing factor 19 | [SwissProt:PRP19_HUMAN] | 55/53 | 6.1/6.8 | x | x | 4/15 | 204 | 81 | 51 |
| 921 | RuvB-like 1 | [SwissProt:RUVB1_HUMAN] | 50/50 | 6.0/6.8 | x |  | 8/16 | 591 | 131 | 50 |
|  | Protein DEK | [SwissProt:DEK_HUMAN] | 43/50 | 8.7/6.8 | x |  | 2/4 | 162 | 92 | 12 |
| 924 | Heterogeneous nuclear ribonucleoprotein H | [SwissProt:HNRH1_HUMAN] | 49/49 | 5.9/6.4 | x |  | 8/15 | 440 | 80 | 53 |
| 926 | Heterogeneous nuclear ribonucleoprotein H | [SwissProt:HNRH1_HUMAN] | 49/49 | 5.9/6.3 | x |  | 8/12 | 421 | 87 | 44 |
| 941 | RuvB-like 2 | [SwissProt:RUVB2_HUMAN] | 51/48 | 5.5/6.1 | x |  | 9/15 | 412 | 70 | 42 |
|  | Spliceosome RNA helicase BAT1 | [SwissProt:UAP56_HUMAN] | 49/48 | 5.4/6.1 | x |  | 2/12 | 73 | 38 | 36 |
|  | ATP-dependent RNA helicase DDX39 | [SwissProt:DDX39_HUMAN] | 49/48 | 5.5/6.1 | x |  | 2/8 | 73 | 38 | 19 |
| 954 | Histone-binding protein RBBP7 | [SwissProt:RBBP7_HUMAN] | 48/47 | 4.9/5.4 | x |  | 5/5 | 244 | 84 | 13 |
| 961 | Alpha-enolase | [SwissProt:ENOA_HUMAN] | 47/46 | 7.0/7.6 | x | x | 9/15 | 544 | 86 | 57 |
| 964 | Alpha-enolase | [SwissProt:ENOA_HUMAN] | 47/46 | 7.0/7.2 | x | x | 9/13 | 526 | 107 | 50 |
| 965 | Alpha-enolase | [SwissProt:ENOA_HUMAN] | 47/46 | 7.0/6.9 | x |  | 6/12 | 163 | 44 | 44 |
| 986 | Proliferation-associated protein 2G4 | [SwissProt:PA2G4_HUMAN] | 44/45 | 6.1/6.6 |  | x | 3/13 | 149 | 62 | 46 |
| 989 | Proliferation-associated protein 2G4 | [SwissProt:PA2G4_HUMAN] | 44/45 | 6.1/6.8 | x | x | 4/12 | 130 | 65 | 44 |
| 990 | 26S protease regulatory subunit 7 | [SwissProt:PRS7_HUMAN] | 49/45 | 5.7/6.4 | x |  | 6/15 | 369 | 96 | 39 |
| 1093 | Heterogeneous nuclear ribonucleoprotein D0 | [SwissProt:HNRPD_HUMAN] | 38/39 | 7.6/8.4 | x |  | 2/6 | 34 | 18 | 13 |
| 1113 | Poly(rC)-binding protein 1 | [SwissProt:PCBP1_HUMAN] | 37/38 | 6.7/7.4 | x | x | 9/14 | 340 | 56 | 77 |
| 1115 | Mitotic checkpoint protein BUB3 | [SwissProt:BUB3_HUMAN] | 37/38 | 6.4/7.1 | x |  | 8/14 | 310 | 59 | 55 |
| 1191 | Serine/threonine-protein phosphatase PP1-beta catalytic subunit | [SwissProt:PP1B_HUMAN] | 37/34 | 5.8/6.1 | x |  | 2/10 | 62 | 41 | 35 |
| 1278 | Putative heterogeneous nuclear ribonucleoprotein A1-like protein 3 | [SwissProt:RA1L3_HUMAN] | 34/30 | 9.2/8.0 | x |  | 5/7 | 249 | 96 | 28 |
| 1285 | Heterogeneous nuclear ribonucleoprotein A1-like protein | [SwissProt:ROA1L_HUMAN] | 34/30 | 9.1/7.8 | x |  | 4/7 | 169 | 68 | 24 |
| 2039 | Histone-binding protein RBBP4 | [SwissProt:RBBP4_HUMAN] | 48/48 | 4.7/5.1 | x |  | 7/10 | 414 | 103 | 27 |

^#^ Number of unique identified peptides in MS/MS and in MS+MS/MS searches. dcSSc: diffuse cutaneous SSc; lcSSc limited cutaneous SSc; MW: molecular weight (kDa); SSc: systemic sclerosis; th/es: theoretical/estimated.
